# Supplementary material for: High risk oral contraceptive hormones do not directly enhance endothelial cell procoagulant activity in vitro
Source: PLoS One. 2023 Apr 19;18(4):e0284333. doi: 10.1371/journal.pone.0284333 (PMC10115293; doi:10.1371/journal.pone.0284333)
Supplement: S1 File — (DOCX) [file pone.0284333.s001.docx]

**SUPPLEMENTARY MATERIALS for “High risk oral contraceptive hormones do not directly enhance endothelial cell procoagulant activity”**

Emma G. Bouck, Marios Arvanitis, William O. Osburn, Yaqiu Sang, Paula Reventun, Homa K. Ahmadzia, Nicholas L. Smith, Charles J. Lowenstein, Alisa S. Wolberg

**SUPPLEMENTAL TABLES**

**Supplemental Table 1. RT-qPCR primer sequences.**

| **Gene** | **Forward primer** | **Reverse primer** |
| --- | --- | --- |
| *ESR1* | GGC ATT CTA CAG GCC AAA TTC | GGC AGA TTC CAT AGC CAT ACT |
| *ESR2* | TGG GCA CCT TTC TCC TTT AG | AGG TGT GTT CTA GCG ATC TTG |
| *F3* | AAG CAC TGT TGG AGC TAC TG | GGG TCT TCA TGC TCC GAA ATA |
| *F8* | CCT CTC CTC ATC TGC TAC AAA G | CCA GCT TCG GTT CTC ATC AA |
| *TFPI* | ACA GAC AGC AGC GAC TTT AG | CAA GAA ACT GGC GAT TGA AGA G |
| *THBD* | GGG TGT GTC TGC TCA GTA AT | CTG ACT TGG CCT GCT ACT TAT |
| *ITGAV* | AGC CCA GTT GTA TCT CAC AAA G | CCC AGT TTG GAA TCG GAA GAA |
| *ITGB3* | TGC CTT ATT GGC AGC TCT AC | GCA GTG AGG GTG TGG AAT TA |
| *PLAT* | CCA AGG TTA CCA ACT ACC TAG AC | GAA GAG GCG GGA TCT CAT TT |
| *PGR* | ATT ACC AGT GTT CCC GTC TTC | CCT GTA CTT CCT CCA GCA TAA G |
| *SERPINE1* | GCC CTT GAG TGC TTG TTA GA | TGG CTG GAC TTC CTG AGA TA |

Additional supplementary tables are available in the associated Excel file (Supplemental Materials 2).

**SUPPLEMENTAL FIGURES**

**
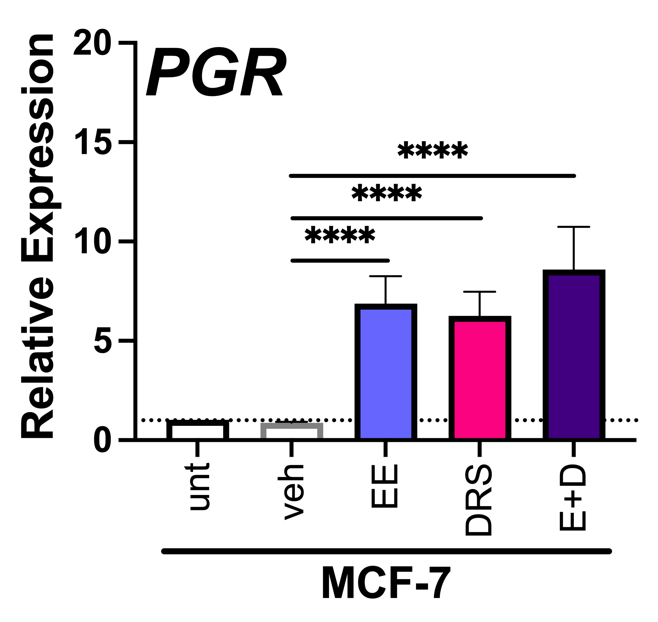
**

**Figure S1. EE and drospirenone have transcriptional activity *in vitro.*** MCF-7 cells were treated with vehicle (0.7% ethanol), 17β-estradiol (1 nM), EE (1 nM), drospirenone (DRS; 100 nM), or EE and drospirenone (E+D) for 24 hours before extracting RNA. *PGR* transcripts were measured by RT-qPCR and normalized to the untreated control. N=7; Bars = mean + SEM; ****p<0.0001

**
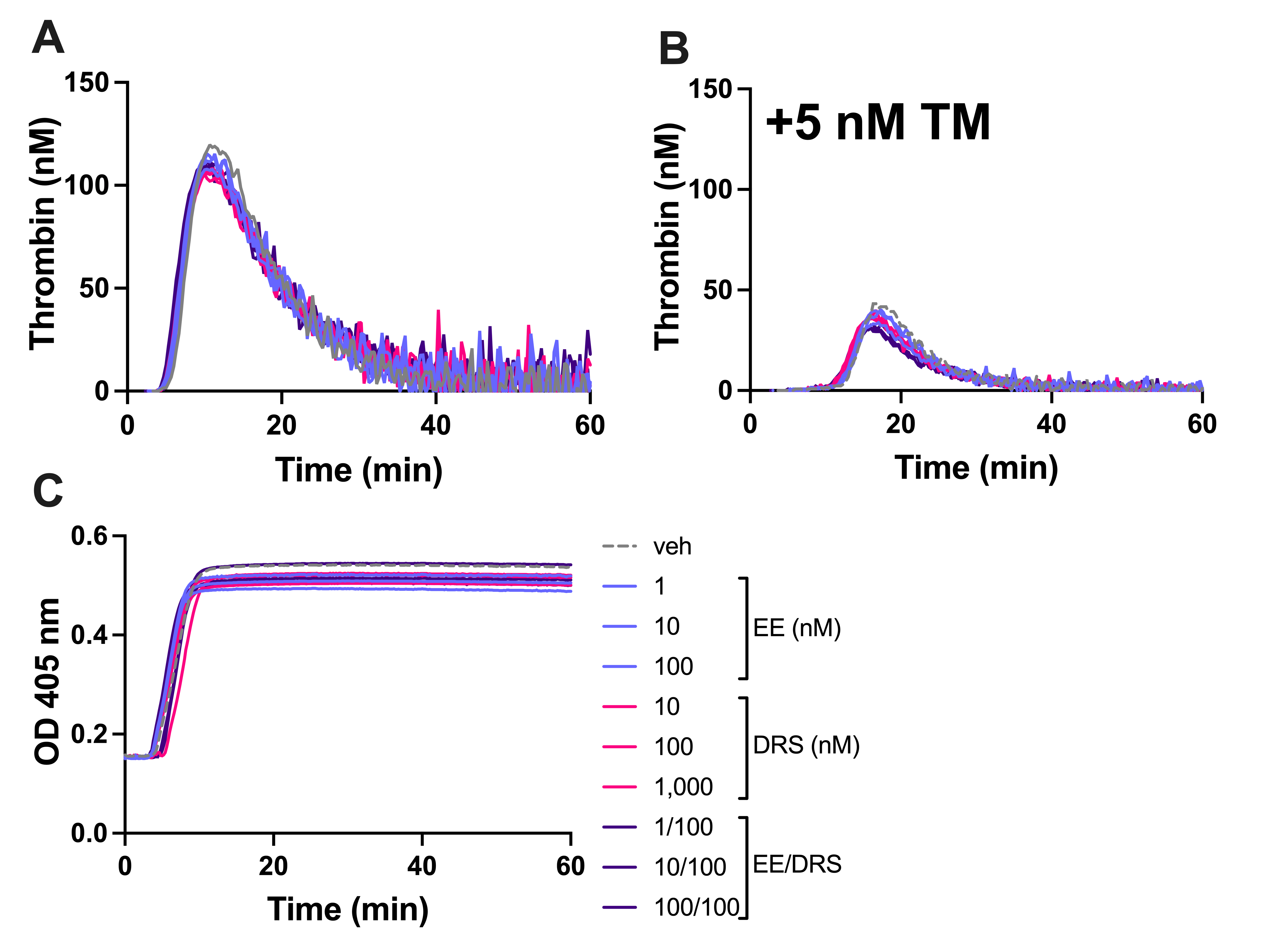
**

**Figure S2.** **EE and drospirenone do not change plasma thrombin generation or fibrin formation.** Vehicle (0.7% EtOH), EE and/or drospirenone were added to NPP at the indicated final concentrations. Thrombin generation was initiated with 1 pM tissue factor, phospholipids, and calcium in the **(A)** absence or **(B)** presence of 5 nM thrombomodulin (TM). **(C)** Fibrin formation was initiated similarly and measured by turbidity. Curves are representative of N=3 experiments performed in duplicate.

**
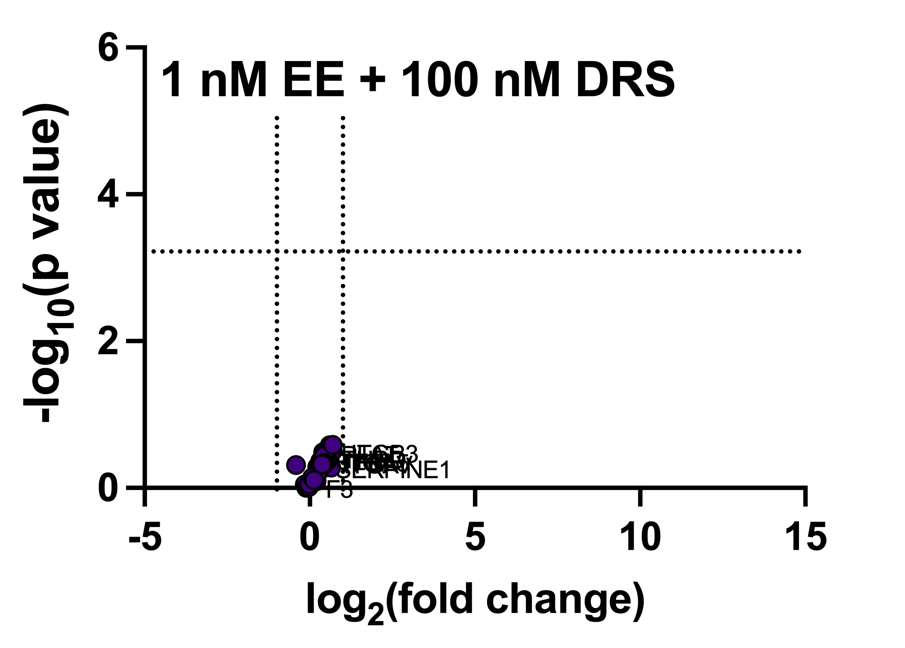
**

**Figure S3. EE and drospirenone do not alter transcription across an endothelial-specific 84-gene panel in HDMVEC.** Primary HDMVEC from a separate donor (Lot 19TL136480) were treated for 24 hours with 1 nM EE and 100 nM drospirenone. RNA expression was measured by RT-qPCR using Qiagen’s RT^2^ Profiler PCR Array and compared to untreated control to determine fold-changes. The same genes evaluated in Figure 1-2 are labeled. P-value cutoff was determined by ɑ=0.05/84 tests.


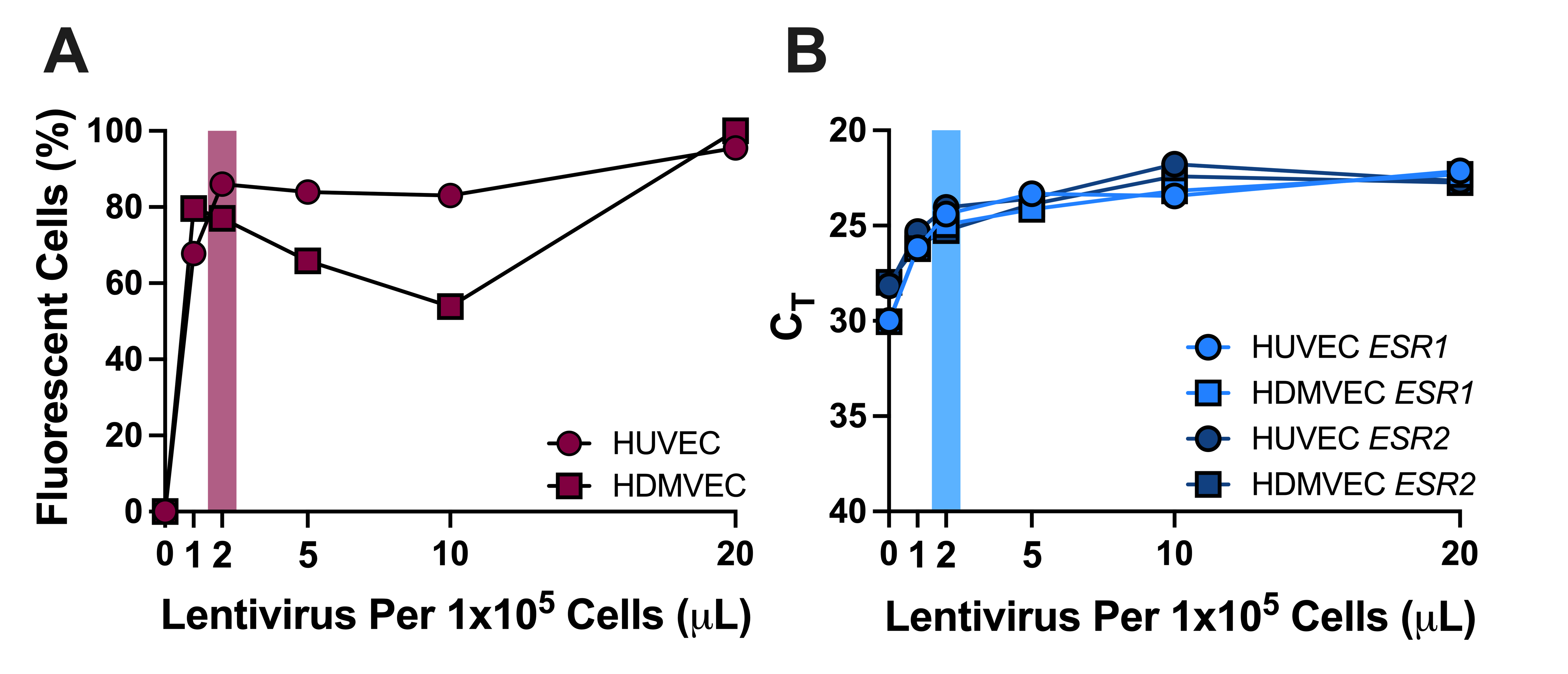


**Figure S4. Optimization of lentiviral titer.** HUVEC and HDMVEC were seeded in 6-well plates at 1x10^5^ cells/well and infected with 1-20 μL of lentiviral particles harvested from HEK293T cells. **(A)** After 72 hours, lenti-mCherry-transduced cells were dissociated and quantified by bright field and fluorescent microscopy. The number of mCherry foci per field was divided by the total number of cells observed in the same field. **(B)** RNA was extracted from lenti-*ESR1* and -*ESR2-*infected cells to measure *ESR1* and *ESR2* expression. Raw C_T_ values were used to determine the optimal lentiviral dose to maximize transcript overexpression while minimizing lentiviral dose.


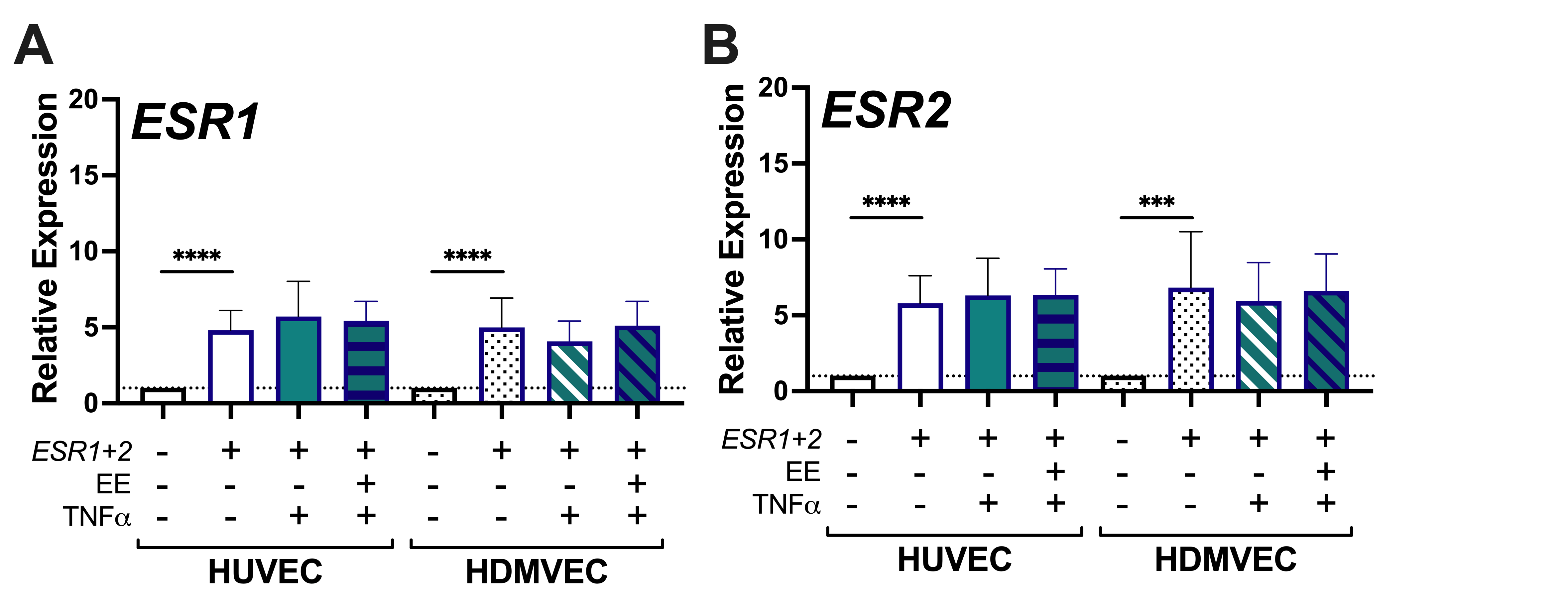


**Figure S5. TNFɑ does not alter *ESR1* or *ESR2*** **expression by ECs.** Expression of **(A)** *ESR1* and **(B)** ESR2 was measured in HUVEC and HDMVEC treated with lenti-ESR1+2 for 72 hours followed by 10 ng/mL TNFɑ and 1 nM EE for 24 hours (N=9-10; Bars = mean + SEM; ***p<0.001, ****p<0.0001).
