# Supplementary figures and images for: High risk oral contraceptive hormones do not directly enhance endothelial cell procoagulant activity in vitro
Source: PLoS One. 2023 Apr 19;18(4):e0284333. doi: 10.1371/journal.pone.0284333 (PMC10115293; doi:10.1371/journal.pone.0284333)

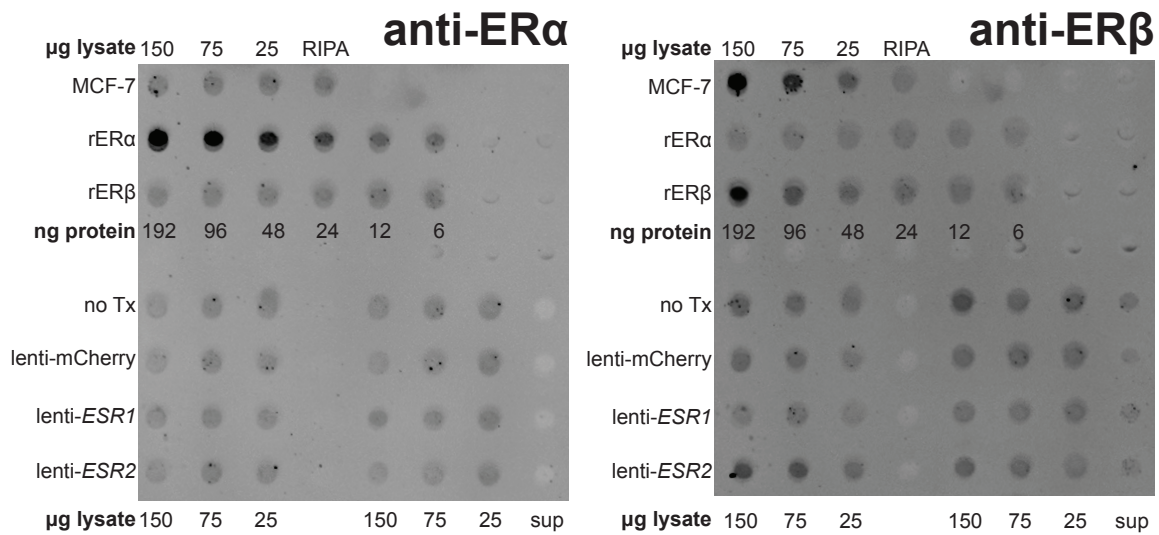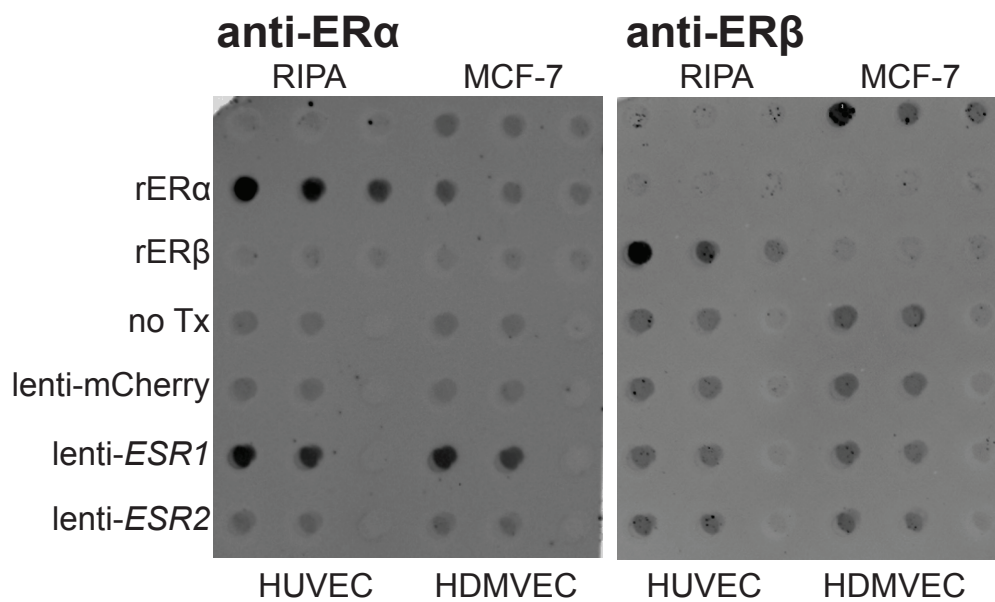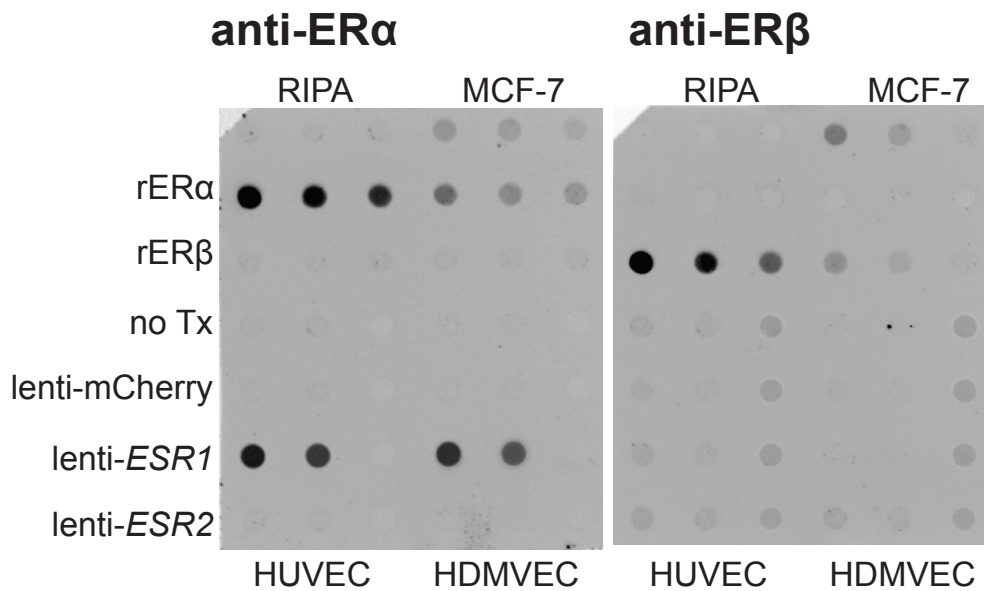

Supplement: S3 File — (PDF) [file pone.0284333.s003.pdf]
